# Graph attention networks based multi-agent path finding via temporal-spatial information aggregation

**DOI:** 10.1371/journal.pone.0318981

**Published:** 2025-06-16

**Authors:** Qingling Zhang, Peng Wang, Cui Ni, Xianchang Liu

**Affiliations:** School of Information Science and Electrical Engineering, Shandong Jiaotong University, Jinan, China; Zhejiang Normal University, CHINA

## Abstract

An effective Multi-Agent Path Finding (MAPF) algorithm must efficiently plan paths for multiple agents while adhering to constraints, ensuring safe navigation from start to goal. However, due to partial observability, agents often struggle to determine optimal strategies. Thus, developing a robust information fusion method is crucial for addressing these challenges. Information fusion expands the observation range of each agent, thereby enhancing the overall performance of the MAPF system. This paper explores a fusion approach in both temporal and spatial dimensions based on Graph Attention Networks (GAT). Since MAPF is a long-horizon, continuous task, leveraging historical observation dependencies is key for predicting future actions. Initially, historical observations are fused by incorporating a Gated Recurrent Unit (GRU) with a Convolutional Neural Network (CNN), extracting local observations to form an encoder. Next, GAT is used to enable inter-agent communication, utilizing the stability of the scaled dot-product aggregation to merge agents’ information. Finally, the aggregated data is decoded into the agent’s final action strategy, effectively solving the partial observability problem. Experimental results show that the proposed method improves accuracy and time efficiency by 24.5%, 47%, and 37.5%, 73% over GNN and GAT, respectively, under varying map sizes and agent densities. Notably, the performance enhancement is more pronounced in larger maps, highlighting the algorithm’s scalability.

## 1. Introduction

With the rapid advancement of artificial intelligence, multi-agent technology has seen significant progress and is now widely applied in areas such as warehouse logistics [1–3], inspection [4–6], security, and autonomous driving [7–10]. As computational capabilities improve and algorithms are optimized, multi-agent systems— which simulate the cooperation and competition among agents—have become vital for solving complex challenges. In such systems, Multi-Agent Path Finding (MAPF) plays a critical role in enabling autonomous navigation for mobile agents. The primary goal of MAPF is to generate efficient paths for multiple agents within given constraints, ensuring they can safely move from the starting point to the target while avoiding collisions with obstacles or other agents [11–13]. The effectiveness of MAPF directly influences the operational efficiency of multi-agent systems. Although path finding techniques have received much attention, there is still a partial observability problem in MAPF. Since each intelligence can only observe the information within its field of view, this may lead to the problem of “short-sightedness” of agents in complex scenes, which may lead to collisions. In order to solve this problem, we extend the observable range of each agent and realize the information sharing between agents, so as to improve the scientific decision-making and the reliability of path finding.

In centralized MAPF, a master control unit usually grasps all information environment and controlled agent information, uses planning algorithm to decompose tasks, and then distributes them to each controlled agent to organize them to complete tasks. Search-based algorithms [14,15], conflict-based search algorithms [16,17], cost-growth tree search algorithms [18] and statute-based algorithms [19] are the four categories into which representative centralized MAPF algorithms can be separated. The centralized MAPF algorithm is a more classical class of MAPF algorithms, which can achieve better results in terms of the speed and quality of the solution, but its flexibility and adaptability to the environment are poor.

Compared to centralized MAPF, distributed MAPF allows multiple agents to learn concurrently, leading to higher learning efficiency [20], as well as greater flexibility and adaptability to the environment. Currently, most distributed MAPF algorithms are based on reinforcement learning [21–24]. However, when applied to multi-agent path finding, reinforcement learning faces challenges such as complex state-action combinations, slow learning speeds, and sparse rewards. To address these issues, imitation learning [25–27] has been proposed as a solution, using expert algorithms and providing dense rewards to tackle multi-agent path finding problems. Additionally, as agent density increases, effective communication [28,29] mechanisms are needed for agents to share position and path information, enabling coordination in path finding and avoiding conflicts. Many Graph Neural Network (GNN)-based communication methods fail to account for the relative importance of features received from neighboring agents, which affects information fusion. As a result, agents may fail to assess the intentions of neighboring agents, leading to poor decision-making that impacts the Accuracy and Time of path finding.

In this paper, we study to propose a method based on temporal-spatial two-dimensional information fusion based on the idea of imitation learning on the basis of GAT. The method utilizes the attention of scaled dot-product to realize the communication of agent, and fuses the historical information and current observation information among multiple agents, focusing on solving the partial observability problem of agents, with a view to improving the Accuracy and Time of MAPF under different size maps and different agent densities. The main contributions of this paper are as follows:

(1) Considering that MAPF is a continuous long-view task, the gated recurrent unit (GRU) is added to the feature extraction network (CNN), while the features of the agent’s historical observation information and the current observation information are extracted and fused to improve the observability of the agent in the time dimension;
(2) GAT is used to establish message communication between multiple agents, and the scaled dot-product attention is used to assign importance weights to the features received from neighboring agents, which can improve the efficiency of information aggregation, and thus improves the observability of agents in the spatial dimension;
(3) Experimental results demonstrate that our model can achieve better results in both Accuracy and Time of MAPF, especially on large-scale maps with high agent density.

## 2. Related work

### 2.1. Path finding based on historical information

In recent years, many researchers have used Recurrent Neural Networks (RNN) to encode historical trajectories, capturing key patterns in time series data [30,31], especially when historical information needs to be considered and future states need to be predicted. By modeling previous paths, motion history, and environmental changes, RNNs can help agents predict the optimal paths to avoid obstacles or adapt to different working scenarios. For example, Wang et al. [32]. used Long Short-Term Memory (LSTM) networks to manage local path trajectories, focusing on extracting historical trajectories to reduce path duration and length. El–Ela et al. [33] combined historical and real-time data with LSTM to predict the next optimal path segment using a neural network model. Compared to LSTM, the Gated Recurrent Unit (GRU) structure is simpler, faster in computation, requires fewer parameters, and is more suitable for resource-constrained or real-time applications. Feng et al. [34], Huang et al. [35] and Choi et al. [36] all applied GRU to the multi-agent path finding problem. To capture more comprehensive entity information from neighboring entities and relationships, Tiwari P et al. [37] proposed a GRU-based method that takes into account the memory of relationships in the path. Ling-Xiao et al. [38] proposed a GRU-GAT framework aimed at solving the issue of preserving neighbor information and historical trajectory information.

### 2.2. Communication-based path finding

In multi-agent path finding, communication allows for the sharing of position information, task status, and environmental data, which helps avoid path conflicts, optimize overall path finding, and enables real-time adjustments in dynamic environments [28,29]. Li et al. [39] used Convolutional Neural Networks (CNN) to extract sufficient features from the local observations of each agent and employed Graph Neural Networks (GNN) [40,41] to transmit these features between agents. They then trained the model using imitation learning based on expert algorithms. However, this approach does not consider the relative importance of the features received from neighboring agents before making decisions. Building on this, Li et al. proposed the Message-aware Graph Attention Network (MAGAT) [42] in 2021, which uses a dot-product attention method to determine the importance of features received from each neighboring agent. They further incorporated multi-head attention mechanisms and a bottleneck structure into the MAGAT model to test the communication performance of MAGAT-P and MAGAT-B models, but no significant improvements were observed. Additionally, Lin et al. [43] introduced the SACHA method, which utilizes communication between agents to facilitate information exchange, enabling path finding in crowded environments. Wang et al. [44] also proposed SCRIMP, which relies on an improved Transformer for local communication, assisting in generating independent and conflict-free paths between agents.

## 3. SDPGAT-G infrastructure

**Fig 1:**
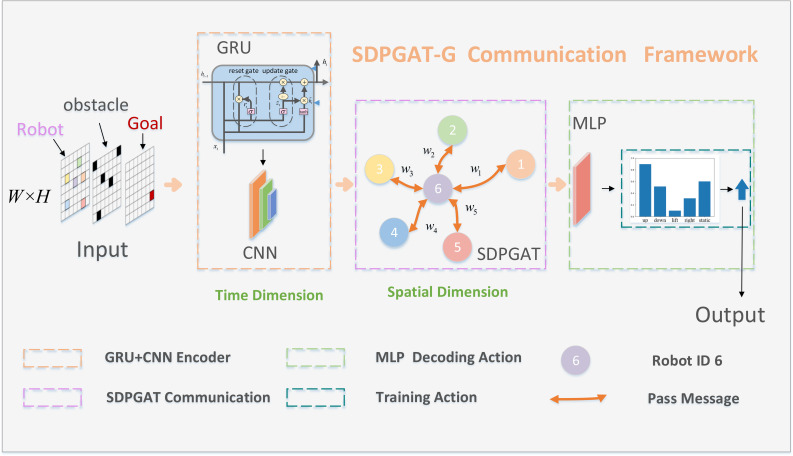
The framework of our model.

Our model proposed in this paper contains three main parts: extracting time dimension features using GRU-CNN, aggregating spatial dimension information using SDPGAT-G, and decoding action strategies using MLP. The model framework is shown in Fig 1.

### 3.1. Formulation of the MAPF problem

Modeling MAPF can transform MAPF into a sequential decision problem. Each agent needs to take an immediate action at time *t* with three constraints: (1) to reach the goal point from the start point quickly; (2) to ensure that the chosen path is optimal; and (3) to avoid collisions between the agents as much as possible [45].

**Fig 2:**
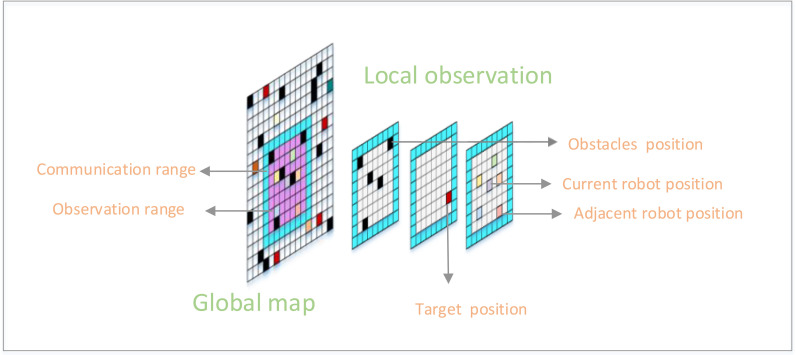
Agent observation range mapping.

Grids map: Firstly, a grid map *M* of the two-dimensional world is generated, as shown in Fig 2, with length and width *W* and *H*, respectively. Where, *M* contains a set of static obstacles *S*, S⊂M. Let $R=\left\{R_{1},R_{2},\cdots R_{N}\right\}$ be a set of *N* agents, each agent having an independent start point $P_{S}$ and a goal point $P_{G}$, respectively.

Observation range: In the grid map *M*, agent *i* has a local field of view FOV defined by radius $r_{FOV}^{i}$ (the purple range in Fig 2), beyond which the agent observes nothing of size $W_{FOV}\times H_{FOV}$. The agent is located at the center of the local field of view, it is not aware of the global position it is in, and the map perceived by agent *i* is defined as $O_{t}^{i}\in R^{W_{FOV}\times H_{FOV}}$. To simplify the learning task of MAPF, in a finite field of view, we separate the available information into different channels. Specifically, the feature tensor $O_{t}^{i}$ observed by agent *i* at time *t* consists of a binary-valued matrix representing the position of the obstacle, the target point, the position of the agent (if it is within the FOV), and the positions of other observable agents, respectively, as shown in Fig 2.

Purple ranges indicate observation ranges, blue ranges and within indicate communication ranges, black squares indicate obstacles, colored squares indicate each agent, and red squares indicate target points.

When agent is close to the edge of the map *M*, obstacles are added to all positions outside the observation boundary FOV. When the agent’s target point is not within range $r_{FOV}^{i}$, the target point information is mapped on its observation range boundary and the agent only perceives the direction of the target.

Communication: Each agent can only communicate with neighboring (within the communication range COM) agents, each agent has a communication radius $r_{COM}^{i}$, beyond the size of the range of $W_{COM}\times H_{COM}$ (the blue range shown in Fig 2), the communication between the agents cannot be carried out. Assuming that such communication is formalized in terms of a dynamic distance-based communication network, the graph $G_{t}$ is defined as the communication network $G_{\text{t}}=(V,E_{t},w_{t})$ of the agent at time *t*, where, v is the set of vertices, i.e., agents, in the graph, $E\subset \left\{\left(V,V\right)|V\in N\right\}$ is the set of edges consisting of vertices, and $w_{t}\to E_{t}$ is the function that assigns weights to the edges. Since the graph is distance-based, $V_{i}$ and $V_{\text{j}}$ can communicate with each other at time *t* when the coordinates of the agents $V_{i}$ and $V_{\text{j}}$ are $\left|{\left(x_{i}-x_{j}\right)}^{2}+{\left(y_{i}-y_{j}\right)}^{2}\right|\leq r_{COM}$, where, $r_{COM}>0$ and $(V_{i,}V_{j})\subset E_{t}$.

Conversely, then $V_{i}$ and $V_{\text{j}}$ cannot communicate. By representing the information of vertices $V_{i}$ and $V_{\text{j}}$ as $\left(V_{i},V_{j}\right)$, the information between the nodes of the whole graph $G_{t}$ can be stored as a two-dimensional array a[i][j], represented by the adjacency matrix $A_{t}$, as expressed in Eq. (1).


$$A_{t}=a[i][j]= \{\begin{array}{c} \begin{array}{cc} 1 & \text{if (i,j)} \end{array}\in E\\ \begin{array}{cc} 0 & \text{Otherwise} \end{array} \end{array})$$ (1)

The corresponding edge weight $w_{t}(V_{i},V_{j})=w_{t}^{ij}=A_{ij}E_{ij}$, i.e., $A_{ij}$ denotes the communicability between agents and $E_{ij}$ denotes the importance of communication between agents.

### 3.2. Temporal-dimensional information extraction by GRU-CNN

MAPF is a continuous long-view task type, so it is an important aspect to consider the use of agent’s historical observation information to capture long time continuous features in the time dimension. CNN [46] is a commonly used model for extracting features, which is able to successfully capture the temporal dependency of the observed feature values through the use of filters and convolutions, and thus apply a closer connection between the observed features. Tighter connection between features is applied to MAPF. The main goal of CNN is to aggregate the information of all neighboring pixels to highlight the most important pixels. However, CNN only utilizes the feature information of the agent state at the current moment when performing feature extraction, and does not make use of whether and how much the information of the agent state at the previous moment has any influence on the agent state at the current moment, which may cause the agent to collide or generate suboptimal paths throughout the path finding process due to the incomplete information mastery and collision. Therefore, utilizing historical information [47–49] can help the agent better understand the dynamics of the environment and the actions of other agent by considering multiple time steps in order to optimize the overall path.

GRU [50] is a variant of recurrent neural network, similar to LSTM [51] (Long-Short Term Memory), which is proposed to solve the long-term memory problem. GRU network can effectively reduce the number of parameters of LSTM network units and shorten the training time of the model by optimizing the three gate functions of LSTM, setting forgetting gates and input gates into a single updating gate, and at the same time mixing the neuron states and hidden states. The number of parameters of LSTM network units and shorten the training time of the model. In this paper, GRU is utilized to capture long-term dependencies of information observed by the agent, and gates are effectively utilized to control the flow of information and “memorize” historical information for use in predicting future movement trends. In this paper, GRU is utilized to capture the long-term dependencies of information observed by the agent, and gates are effectively utilized to control the flow of information and to “memorize” historical information that can be used to predict future movement trends.

In the MAPF model, it is assumed that the feature size extracted by each agent at time *t* is *F*. The observations of each agent ${\tilde{O}}_{t}^{i}\in {\mathbb{R}}^{F},i=1,2...,N$ together form the observation matrix $O_{t}$ of all agents, as shown in Eq. (2).


$$O_{t}=\left[\begin{array}{c} {\left({\tilde{O}}_{t}^{1}\right)}^{T}\\ \vdots \\ {\left({\tilde{O}}_{t}^{N}\right)}^{T} \end{array}\right]=\left[O_{t}^{1}\cdots O_{t}^{F}\right]$$ (2)

The feature captured by the CNN is defined as a linear combination of the observation matrix $O_{t}$ of the current agent at the moment *t* and the matrix $A_{t}$ of the neighboring nodes in the graph $G_{t}$. The value of the feature *F* at the node $V_{i}$ at the moment *t*, after the $A_{t}O_{t}\in {\mathbb{R}}^{N\times F}$ operation, is shown in Eq. (3).


$$\Psi (O_{t};A_{t})=\sum_{K=0}^{K-1}A_{t}^{K}O_{t}B_{K}$$ (3)

Where, $\Psi (O_{t};A_{t})$ denotes a linear combination of $O_{t}$ and $A_{t}$, and $B_{K}$ is a set of F×G matrices representing filter coefficients combining different observations, where, *F* and *G* denote the dimensions of the input and output layers of the graph convolution. In addition, $A_{t}^{K}O_{t}=A_{t}^{K}(A_{t}^{K-1}O_{t})$ is computed by exchanging *K* communication exchanges with 1-hop neighbors. The CNN module is composed of a cascade of *L* layer graphical convolutions, each as shown in Eq. (4), followed by an activation function σ:ℝ→ℝ.


$$O_{l}=\sigma [B_{l}(O_{l-1};A)]\begin{array}{cc} & l=1,2,\ldots ,L \end{array}$$ (4)

Where, *σ* acts on each layer of the graph convolution, fusing the output features $O_{l-1}\in {\mathbb{R}}^{N\times F_{l-1}}$ of the previous layer l−1 as input to the current *l* layer and computing the current aggregation information $F_{l}$.

The input-output structure of GRU is similar to that of ordinary RNN [52], which consists of a feature input $O_{t}^{l}$ from the previous *l* layers at the current moment *t* and a hidden state information $h_{t-1}$ from the previous moment existed in the memory, vector splicing, respectively, with its weight vectors to obtain the reset gating $r_{t}$ and update gating $z_{t}$ by performing the dot product operation to get the gating signals after. The reset data is $h^{'}t-1=h_{t-1}\times r_{t}$, and then the reset data $h^{'}t-1$ is spliced with $O_{t}^{l}$, and then the tanh activation function is used to obtain ${\tilde{h}}_{t}$, as shown in Eq. (5).


$$\tilde{h}=\phi (W_{\tilde{h}}\cdot [r_{t}\times h_{t-1},x_{t}])$$ (5)

${\tilde{h}}_{t}$ contains the current input data $O_{t}^{l}$, and the current hidden state is added purposefully, which is equivalent to memorizing the current moment. The updated $h_{t}$ is the sum of the “forgotten” and “memorized” parts, as shown in Eq. (6).


$$h_{t}=(1-z_{t})\times h_{t-1}+z_{t}\times {\tilde{h}}_{t}$$ (6)

Where, $(1-z)\times h_{t-1}$ represents the selective forgetting of some unimportant information in the original hidden state, and $z\times {\tilde{h}}_{t}$ represents the selective “remembering” of some information in the current information node.

It is the memory role of the GRU that enables the agent to utilize its historical observation information and then combine it with the current observation information to coordinate the agent in a longer view of the whole situation. Therefore, in this paper, GRU and CNN are fused into a network structure GRU-CNN, which comprehensively utilizes historical information instead of one-sidedly utilizing only the current information, so that agent $V_{i}$ communicates with other agents through the communication network $G_{t}$ at the moment *t*, thus ensuring that the agent can move to the destination in the shortest possible time while avoiding collisions with other agents. While avoiding collisions with other agents. Utilizing GRU-CNN network for information extraction, Eq. (3) should be changed to Eq. (7).


$$\Psi (O_{t};A_{t};H_{t})=\sum_{K=0}^{K-1}A_{t}^{K}O_{t}H_{t}B_{K}$$ (7)

Where, $H_{t}$ is the history information matrix of the agent’s current moment *t*, which represents the sum of the agent’s memorized history information from moment t=1 to t−1, and is calculated as shown in Eq. (8), *Φ* is the mapping relationship between the current information and the history information.


$$H_{t}=\sum_{t=1}^{T}\Phi (O_{t-1},H_{t-1})$$ (8)

**Fig 3:**
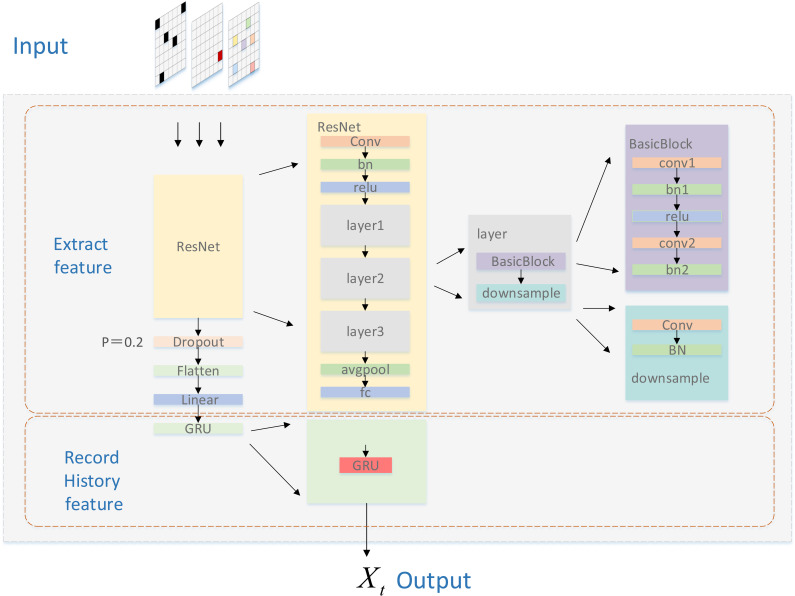
Structure of GRU-CNN feature extraction network.

The new feature $\Psi^{\prime }(O_{t};A_{t};H_{t})$ extracted by GRU-CNN is cascaded to the new output feature tensor $X_{t}$, and the captured information is used for the aggregation of GAT information, the structure of the feature extraction network is shown in Fig 3.

### 3.3. Spatial-dimensional information aggregation and update by SDPGAT-G

GRU-CNN performs feature extraction on the input tensor to obtain the feature tensor $X_{t}=GRU-CNN(O_{t})$. Then in the spatial dimension, GAT is utilized to establish a communication mechanism among multiple agents and the feature tensor $X_{t}$ is passed into the GAT, and then the observability of the agents is improved by aggregating the information of the neighboring agents. However, as the number of agents increases and the feature tensor data dimension becomes higher, GAT uses dot product attention for information aggregation, which may cause the distribution of attention weights to be biased, resulting in the GAT model may be more inclined to focus on some specific dimensions, which will reduce the efficiency of MAPF. Compared with general dot product attention, scaled dot-product attention scales the feature tensor during dot product computation, which can control the dot product value in a smaller range, thus ensuring that the attention weights can maintain a uniform distribution in different dimensions. Especially for large-scale datasets or high-dimensional data, scaled dot-product attention usually has higher computational efficiency and stability.

#### 3.3.1. Information aggregation by SDPGAT-G.

**Fig 4:**
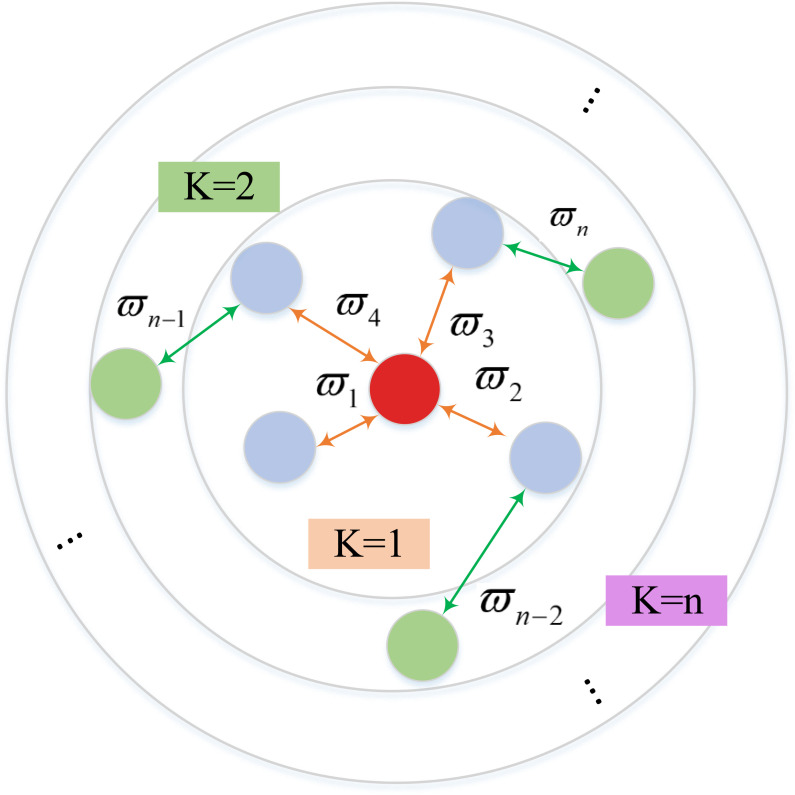
Diagram of SDPGAT-G communication model.

Inspired by the message network MAGAT [42], this paper uses a scaled dot-product attention mechanism as the information aggregation model, such that the edge weights between nodes $W_{t}(V_{i},V_{j})\in \left[0,1\right]$ are determined by the relative importance of the node’s features, which allows the agent to aggregate the information features received from its neighbors with a selective focus. Formally, inspired by the literature [53], a SDPGAT-G model is defined, as shown in Fig 4.

The red circle is the current agent, the blue circle is the current agent’s neighbor within K=1 hop, and the green circle is the blue circle’s K=1 hop neighbor (and also the red circle’s neighbor within K=2 hops)

Assuming that the input feature tensor of the GNN is $X_{\text{t}}$, there is a feature tensor set $X_{t}=\left\{x_{t}^{1},x_{t}^{2},...x_{t}^{N}\right\},x_{t}^{i}\in {\mathbb{R}}^{F}$. Where, *N* is the number of nodes and *F* is the number of features per node, the tensor $X_{\text{t}}$ is a matrix of size N×F. In order to compute the attention mechanism to find the assigned weights and get the corresponding input-to-output transformation relations, it is necessary to consider a trainable matrix $W\in {\mathbb{R}}^{F\times F^{\prime }}$ containing the weights of all nodes, where, *F* and *F*^′^ are the number of input and output features of the matrix *W*, respectively.

The scaled dot-product model is chosen for each vertex to compute the attention score, and $e_{ij}$ is the correlation score between each agent $V_{i}$ and its neighbor agent $V_{j}$, so that each agent gets a correlation score of the same dimension. Let η(x,y) be the function to calculate the importance between *x* and *y*, which represents the importance of the influence of the information of the neighbor agent $V_{j}$ on the decision making of the agent $V_{i}$, and $d_{x}$ is the dimension of the input feature tensor value $X_{\text{t}}$, then $e_{ij}$ is calculated as shown in Eq. (9). The reason for dividing by $\sqrt{d_{x}}$ is to limit the value of the attention score to an appropriate range, which facilitates model optimization, improves the stability of the network during training, and ensures that the weights are uniformly distributed in all dimensions.


$$e_{ij}=\frac{\eta (WX_{t}^{i},WX_{t}^{j})}{\sqrt{d_{x}}}$$ (9)

After that, masked attention is used to allocate the attention only to the node set $N_{i}$ of agent $V_{i}$, which is the set of all neighboring nodes of the node, $j\in N_{i}$. The attention score $a_{ij}$ is obtained by regularizing the neighboring nodes by Softmax operation as shown in Eq. (10).


aij=softmax(eij)=exp(eij)∑j∈Niexp(eij)=exp(LeakyReLU(eij))∑j∈Ni(LeakyReLU(eij)) (10)

For Eq. (10), the larger the order of magnitude of the attention score $e_{ij}$, the larger the input to the Softmax function, and when normalized, the result will be very close to 1. Softmax will assign almost all the weight to the vertex corresponding to the maximum value, resulting in a biased weight assignment. To solve this problem, we divide the similarity score by the square root of the dimension $d_{x}$ of the feature tensor value $X_{\text{t}}$. In this way, the attention score $e_{ij}$ is obtained as an input with a relatively small gap before Softmax is normalized, ensuring the stability of the attention weight allocation, and more conducive to the next step of message to pass the update within the communication hop count *K*.

#### 3.3.2. Message delivery and update by SDPGAT-G.

The message delivery update between multiple agents is synchronized with the process of agent aggregation of neighbor node information. In this paper, we use $X_{i}^{K-1}\in {\mathbb{R}}^{F}$ to denote the feature tensor of a vertex communicating at the K−1 ^st^ hop, and $s_{ij}$ denotes the attributes of the edges of vertex $V_{i}$ and vertex $V_{j}$. First, the information of the vertex set $N_{i}$ adjacent to vertex $V_{i}$ is aggregated to vertex $V_{i}$ as shown in Eq. (11).


$$X_{N_{i}}^{K}=\phi^{K}(X_{i}^{K-1},X_{j}^{K-1},s_{ij}))$$ (11)

Where, *ϕ* is the differentiable function used to aggregate the neighbor information. The obtained neighbor information $X_{N_{i}}^{K}$ is nonlinearly combined with the attention score matrix $W_{a_{ij}}$ using the *λ* function as shown in Eq. (12).


$$X_{N_{i}}^{w}=\underset{j\in N_{i}}{\lambda }(X_{N_{i}}^{K},W_{a_{ij}})$$ (12)

Finally, the updated information of vertex $V_{i}$ can be obtained by putting the information of vertex $V_{i}$ together with the neighbor information obtained by aggregation through a *γ* nonlinear transformation, as shown in Eq. (13).


$$X_{i}^{K}=\gamma^{K}(X_{i}^{K-1},\underset{j\in N_{i}}{\lambda }\phi^{K}(X_{i}^{K-1},X_{j}^{K-1},s_{ij}),W_{a_{ij}})$$ (13)

The vertex $V_{i}$ (i.e., agent $R_{i}$) synchronously completes message delivery and aggregation with its *K* -hop neighbors when updating its own information, and the weight parameter of GAT enables $V_{i}$ to aggregate more important information more efficiently.

### 3.4. Aggregated information decoding by MLP

In the model of MAPF, the path finding problem is abstracted into a sequential decision problem, where at time *t*, the current problem to be solved by each agent is to reach the destination. The goal of this work is to learn a mapping $Y_{t}$ such that agent $V_{i}$, at time *t*, learns a mapping $Y_{t}$ and determines an appropriate action $a_{t}=Y_{t}(G_{t},O_{t})$ based on the agent’s observed information $O_{t}$ and the information of the communication network $G_{t}$. The MLP is analogous to this mapping $Y_{t}$.

MLP [54] (Multilayer Perceptron), a multilayer, fully connected neural network, is widely used in a number of predictive classification problems. In this paper, by learning the features extracted from the CNN and fused by the GAT, the MLP employs a probability-distributed stochastic action strategy to decode the prediction of all possible actions to be taken for each agent *i*, and then synthesizes to give a current optimal action $a_{t}$, which determines a collision-free path from the starting point to the goal point.

## 4. Experiments

### 4.1 Experiment target

The proposed algorithm in this paper is trained and tested using randomly generated map sets and compared with GNN [39], GAT [55], MAGAT [42], MAGAT-P4, MAGAT-P4-B, RL-RVO [30], ERL-MAPF [31] for evaluating the effectiveness of the algorithm under different map sizes and agent densities. GNN is a graph neural network model that enables communication between multiple agents, but it does not weigh the relative importance of features received from neighboring agents and is prone to collision when the number of agents increases; GAT is a graph attention network that performs a weighted summation of individual nodes, but the attention is not stable enough in this way; and MAGAT, also a graph attention neural network model, uses dot product summation to assign weights. Using dot product summation to assign weights, but this calculation leads to biased weights; MAGAT-P4 and MAGAT-P4-B are both variants based on MAGAT, but with better performance; RL-RVO is a path finding algorithm based on feature extraction and action computation by GRU in continuous space; ERL-MAPF is an evolutionary algorithm based on GRU in the MAPF method.

### 4.2. Experimental setup

The CPU used for our experiments is Inter(R) Core(TM) i7-10700, the GPU is NVIDIA Corporation Device 2482, and the operating system is ubuntu20.04. The experimental environment was implemented with the help of Tensorflow2.2.0. The specific experimental parameter settings are shown in Table 1.

**Table 1:** Experimental parameter settings.

parameter	Value	explanation
**F**	128	CNN/GRU feature dimensions
**G**	3	Number of Graph Filter Tap
**ks**	3 × 3	kernel sizes
**p**	0.2	dropout rates
**l**	3	SDPGAT layers
**K**	2	communication hop
**Adam**	200 ~ 300	Adam optimizer
**Lr**	$10^{-3}$ ~ $10^{-6}$	Learning rate
**max_epoch**	300	Training Max epoch
**save_epoch**	4	Training save epoch
**num_testset**	4500	The number of test
**commR**	7	Communication radius
**FOV**	5	Field of View radius
**weight_decay**	$10^{-5}$	Weight decay rate

### 4.3. Data preparation

We prepared the test map sets shown in Table 2 with randomly generated obstacles in the maps, each type of map set contains 20000 randomly generated maps. Then we train the model of this paper using Map 1 and test it on all map sets, and the resultant data is the average of multiple tests. Agent density is calculated as shown in Eq. (14).

**Table 2:** Information on each atlas.

Map Number	Map W×H	Agent $n_{R}$	Density *ρ*
**Map 1**	20 × 20	10	0.025
**Map 2**	28 × 28	20	0.025
**Map 3**	50 × 50	60	0.025
**Map 4**	50 × 50	10	0.004
**Map 5**	50 × 50	20	0.008


$$\rho =\frac{n_{R}}{W\times H}$$ (14)

Where W,H is the width and height of the map and $n_{R}$ is the number of agents.

### 4.4. Metrics

(1) Accuracy: Accuracy indicates the ratio of the number of tests in which the agent reaches the target point to the total number of tests, as shown in Eq. (15).


$$Accuracy=\frac{M_{reachGoal}}{M_{test}}$$ (15)

$M_{reachGoal}$ is the number of tests in which the agent successfully reaches the target point from the start point within a certain number of steps and time, and $M_{test}$ is the total number of tests. “Success rate” can be used to measure the success or accuracy of a MAPF task.

(2) MakeSpan: Makespan is the ratio of the time required for all agents to move from the start point to the goal point, as shown in Eq. (16).


$$MakeSpan({△}_{\text{ms}})=\frac{\left|P_{ms}-T_{ms}\right|}{T_{ms}}$$ (16)

Where, $P_{ms}$ is the actual time for all agents to move from the start point to the goal point and $T_{ms}$ is the expected time for all agents to move from the start point to the goal point.

(3) FlowTime: FlowTime is the ratio of the difference between the actual path length and the expected path length, as shown in Eq. (17).


$$FlowTime({△}_{ft})=\frac{\left|P_{ft}-T_{ft}\right|}{T_{ft}}$$ (17)

Where, $P_{ft}$ is the actual executed path length and $T_{ft}$ is the expected path length.

(4) FailedReachGoal: Corresponding to accuracy, FailedReachGoal is the ratio of the number of tests in which the agent did not successfully reach the target point to the total number of tests, as shown in Eq. (18).


$$FailedReachGoal=\frac{M_{FailedReachGoal}}{M_{test}}$$ (18)

Where, $M_{FailedReachGoal}$ is the number of times the agent did not complete the test to reach the target point from the start point within a certain number of steps and time.

(5) Time: the time from the beginning to the completion of agent path finding, i.e., the time taken by all agents to reach the goal point from the start point, which is used to measure the efficiency of MAPF. “Time” is an important index to measure the time efficiency of MAPF task.

In the experimental process, this paper chooses the most representative Accuracy and Time as the evaluation indexes of the model, and analyzes and compares different MAPF models on different maps.

### 4.5. Experiments and analysis

#### 4.5.1. Ablation study and analysis.

For the convenience of description, the models proposed in this paper are denoted as MAGAT-G, SDPGAT and SDPGAT-G. Among them, MAGAT-G uses the GRU-CNN network proposed in this paper for the extraction of features from agent-observed information on the basis of MAGAT; SDPGAT uses a different attention mechanism from that of MAGAT. SDPGAT uses the scaled dot-product attention proposed in this paper; SDPGAT-G uses the GRU-CNN network proposed in this paper to extract features from the information observed by the agent on the basis of SDPGAT.

The test is performed from Maps 1–5, and the results are shown in Table 3. From the Table 3, it can be found that the Accuracy with the addition of GRU will be higher than the baseline model, this is because GRU provides the agent with additional history information, which enables it to optimize the strategy under partial observability by learning the history information and the neighbor information and utilizing them when deciding the action, and improves the Accuracy of agent path finding. This effect is not found to be obvious in Map 1, instead, this advantage is more obvious in Maps 2 and 3, especially in Map 3, where the Accuracy of path finding is improved by 31.17% compared to the model MAGAT. It is not difficult to find that the effect of introducing GRU becomes more obvious as the map size increases. This is because when the map size is small and simple, history information does not have much effect on the long horizon decision. However, as the map size increases, the influence of historical information gradually increases, and the synthesis of historical information and current information can better guide the agent to make more reasonable actions, thus improving the Accuracy of agent path finding.

**Table 3:** Results of ablation experiments.

Map	Model	Accuracy(%)	MakeSpan(%)	FlowTime(%)	FailedReachGoal(%)	Time(S)
**Map 1**	MAGAT-G	91.1	17.7	8.4	8.0	627.83
	SDPGAT	91.3	17.4	8.3	7.9	623.53
	SDPGAT-G	91.5	17.1	8.2	7.5	624.29
**Map 2**	MAGAT-G	86.5	42.4	11.5	13.3	2,787.42
	SDPGAT	85.6	49.5	13.1	14.2	2,128.02
	SDPGAT-G	86.7	49.0	12.5	13.1	2,218.36
**Map 3**	MAGAT-G	78.6	65.1	10.1	21.4	4,553.11
	SDPGAT	79.6	78.7	12.7	20.4	4,214.84
	SDPGAT-G	79.1	78.5	12.2	20.9	4,275.37
**Map 4**	MAGAT-G	91.0	17.8	8.4	8.1	634.37
	SDPGAT	91.3	17.5	8.3	8.0	626.35
	SDPGAT-G	91.6	23.6	8.0	7.4	630.59
**Map 5**	MAGAT-G	95.3	16.1	5.4	4.0	3,343.40
	SDPGAT	95.4	16.9	5.6	4.3	3,167.94
	SDPGAT-G	95.5	16.2	5.4	4.4	3,238.99

However, the Accuracy cannot fully measure the quality of the model, as it only focuses on whether the agent achieves the goal within the given time, ignoring the time cost of execution. Therefore, it is also necessary to use Time to measure the model’s pathfinding time. As shown in Table 3, the SDPGAT-G and SDPGAT models have shorter running times than the MAGAT-G model. This is because the scaled dot-product attention uses a scaling factor to adjust the scale of the dot-product attention scores. The purpose of this is to reduce the bias of the weight values when the dimensionality is large. Due to the more stable numerical properties of scaled dot-product attention, the calculation of the attention mechanism is more scientifically sound, thereby saving multi-agent pathfinding time and improving pathfinding efficiency. Especially on Map 2 and Map 3, the proposed model shows great potential, with pathfinding times reduced by 24.90% and 20.03%, respectively, compared to the MAGAT model. This proves that the proposed model is more time-efficient than other models.

#### 4.5.2. Comparison to state-of-the-art.

Similarly, tests were conducted on Maps 1 - 5, with results shown in (Tables 4–8). The proposed SDPGAT-G model outperforms the MAGAT model in both path finding Accuracy and Time, and also surpasses other improved versions of MAGAT, such as MAGAT-P4 and MAGAT-B, as well as GRU-related RL-RVO and ERL-MAPF algorithms. When tested on Map 1, the Accuracy of our model is comparable to that of the seven models. However, as the map size and the number of obstacles increase, we observe that the Accuracy of all models declines to varying degrees, especially for the GNN and GAT models. This could be due to the fact that GNN and GAT typically encounter performance bottlenecks when handling large-scale, dense environments. As the number of obstacles and the map size increase, the graph density and computational complexity grow exponentially, which impacts the stability of the models. In contrast, the decline in the Accuracy of our model is much slower than that of the seven models. Particularly when expanding to Map 2 (as shown in Table 5), the Accuracy of our model improves to varying extents, while MakeSpan, FlowTime, and Time are all reduced. Moreover, this trend becomes even more pronounced on Map 3 (as shown in Table 6), where the Accuracy reaches 79%. MAGAT, as well as MAGAT-P4 and MAGAT-B, performs relatively steadily, but the Accuracy drops significantly on larger and more crowded maps. This might be because these models did not fully utilize historical information to optimize pathfinding decisions. RL-RVO and ERL-MAPF perform relatively well, but still do not have the advantage of our model.

**Table 4:** Test data for Map 1.

	Accuracy(%)	MakeSpan(%)	FlowTime (%)	FailedReachGoal(%)	Time(S)
**GNN**	89.6	19.9	9.4	9.8	615.04
**GAT**	71.5	39.0	16.9	28.0	797.91
**MAGAT**	90.2	18.7	8.8	9.2	627.44
**MAGAT-P4**	88.7	20.9	9.7	10.7	644.97
**MAGAT-B**	88.2	21.4	9.9	11.3	675.7
**RL-RVO**	90.0	/	/	/	637.25
**ERL-MAPF**	90.8	/	/	/	639.45
**SDPGAT-G**	**91.5**	**17.1**	**8.2**	**7.5**	**624.29**

**Table 5:** Test data for Map 2.

	Accuracy(%)	MakeSpan(%)	FlowTime(%)	FailedReachGoal(%)	Time(S)
**GNN**	78.7	66.1	16.8	21.2	3,103.85
**GAT**	51.2	100	31.7	48.7	4,192.54
**MAGAT**	83.0	49.8	13.0	16.5	2,833.91
**MAGAT-P4**	80.7	51.1	14.8	19.1	2,975.09
**MAGAT-B**	79.3	60.0	15.4	20.6	2,995.57
**RL-RVO**	83.0	/	/	/	2,870.34
**ERL-MAPF**	85.8	/	/	/	2,962.58
**SDPGAT-G**	**86.7**	**49.0**	**12.5**	**13.1**	**2,218.36**

**Table 6:** Test data for Map 3.

	Accuracy(%)	MakeSpan(%)	FlowTime(%)	FailedReachGoal(%)	Time(S)
**GNN**	53.8	100	20.3	46.1	5,664.17
**GAT**	20.7	100	38.1	79.2	6,846.27
**MAGAT**	60.3	100	15.2	39.6	5,270.27
**MAGAT-P4**	55.0	100	16.8	45.0	5,396.01
**MAGAT-B**	57.1	100	17.1	42.8	5,327.81
**RL-RVO**	76.3	/	/	/	4,857.10
**ERL-MAPF**	77.8	/	/	/	5,520.23
**SDPGAT-G**	**79.1**	**78.5**	**12.2**	**20.9**	**4,275.37**

**Table 7:** Test data for Map 4.

	Accuracy(%)	MakeSpan(%)	FlowTime(%)	FailedReachGoal(%)	Time(S)
**GNN**	89.7	19.8	9.2	9.7	753.17
**GAT**	74.2	37.1	16.5	25.3	742.16
**MAGAT**	90.2	18.6	8.7	9.1	630.50
**MAGAT-P4**	88.6	20.9	9.7	10.7	666.31
**MAGAT-B**	87.9	21.6	9.9	11.5	631.06
**RL-RVO**	86.9	/	/	/	652.70
**ERL-MAPF**	90.2	/	/	/	660.25
**SDPGAT-G**	**91.6**	**23.6**	**8.0**	**7.4**	**630.59**

**Table 8:** Test data for Map 5.

	Accuracy(%)	MakeSpan(%)	FlowTime(%)	FailedReachGoal(%)	Time(S)
**GNN**	93.4	16.2	5.6	6.2	3,348.03
**GAT**	82.7	51.7	13.6	16.9	4,122.35
**MAGAT**	94.0	19.6	6.1	5.5	3,378.72
**MAGAT-P4**	92.9	23.6	7.1	6.6	3,488.43
**MAGAT-B**	92.2	24.8	7.2	7.4	3,412.52
**RL-RVO**	92.9	/	/	/	3527.17
**ERL-MAPF**	93.2	/	/	/	3629.16
**SDPGAT-G**	**95.5**	**16.2**	**5.4**	**4.4**	**3,238.99**

#### 4.5.3. Trends in different map sizes and densities.

**Fig 5:**
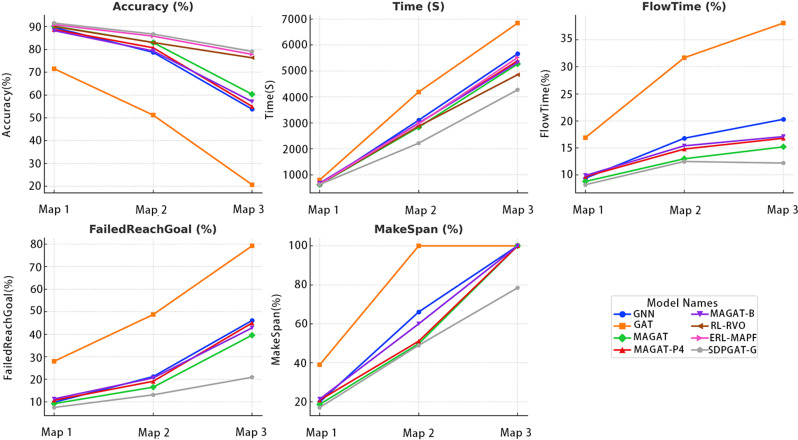
Performance of agents on maps of different sizes.

As the map size increases, as shown in Fig 5, the performance of various algorithms on different metrics undergoes significant changes. For Accuracy, most algorithms maintain a high detection rate on small-sized maps (e.g., Map 1 and Map 2). However, on larger maps (e.g., Map 3 and Map 5), the Accuracy of GAT and GNN declines significantly, possibly due to their failure to fully leverage historical information in path finding, leading to more path conflicts or failures. In contrast, the MAGAT-G and SDPGAT series manage to maintain stable accuracy even on larger maps, demonstrating better scalability in path finding.

Regarding Time and MakeSpan, the impact of large maps on the algorithms is particularly notable. As the map size increases, all algorithms exhibit a certain degree of increase in Time, but the growth rates of SDPGAT-G and RL-RVO are relatively moderate. This suggests that their time complexity performs better when scaling to larger maps. Additionally, large-sized maps are often associated with higher FlowTime, and some algorithms, due to low path-finding efficiency, fail to effectively optimize their paths.

**Fig 6:**
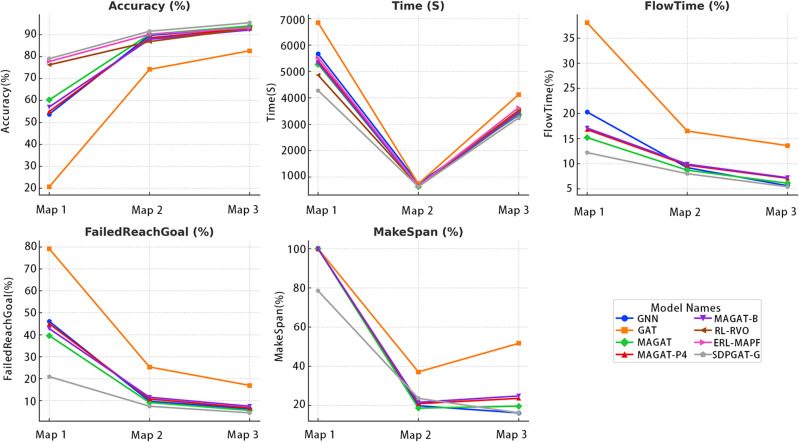
Performance of agents on maps with different densities.

From the perspective of maps with varying densities, as shown in Fig 6, FailedReachGoal serves as a critical indicator. In low-density maps, all algorithms can complete their target tasks effectively with fewer conflicts. However, as the map density increases (e.g., higher obstacle ratios or more agents), the proportion of unfinished goals for GAT and GNN rises significantly. In contrast, superior algorithms, such as the SDPGAT series and MAGAT-G, can still maintain a low failure rate in high-density maps.

In terms of MakeSpan and Time, increasing density significantly elevates the complexity of path finding, causing most algorithms to experience substantial increases in runtime. This is particularly evident for GAT and GNN, which exhibit the largest growth in time overhead and path length. This reflects the computational bottleneck of some traditional methods in complex scenarios. Conversely, algorithms such as SDPGAT-G and ERL-MAPF demonstrate better capabilities in balancing path conflicts and finding efficiency. Even in high-density maps, they manage to maintain shorter total path lengths and lower time costs, making them better suited for multi-agent pathfinding tasks in complex scenarios.

### 4.6. Summary

In summary, the models SDPGAT as well as SDPGAT-G proposed in this paper are able to guarantee the Accuracy and Time of their pathfinding on maps of different sizes, and outperform the baseline model in most cases, which indicates that the SDPGAT family of models has a greater potential in terms of generalization ability and in dealing with congestion to avoid collisions. This proves that the model proposed in this paper is better able to take into account the comprehensiveness of wayfinding, thus ensuring that more agents can complete the wayfinding task in a shorter period of time.

## 5. Conclusion

In this paper, we fuse the observation information of agents in both temporal and spatial dimensions, and propose a scalable and migratory MAPF model, SDPGAT-G. The model utilizes GRU to record the historical information to construct local observation encoders, and uses the SDPGAT mechanism to communicate among agents to improve the MAPF performance. In most cases, all types of evaluation metrics outperform the baseline model. Although our model performs reasonably well in the current experiments, there are still many aspects that need to be further improved, e.g., latency and redundancy problems in the communication process may significantly affect the performance and reliability of the model. These issues are not only related to the real-time performance and accuracy of the model, but may also limit its applicability in dynamic and complex scenarios. In addition, the uncertainty of dynamic environments, diverse scenarios, and complex interactions among agents may pose challenges to the scalability of the model. Therefore, in the subsequent research, we will focus on solving these problems, especially on optimizing the communication redundancy problem, designing more efficient communication topology and data processing mechanisms to enhance the overall performance of the system, and verifying the scalability of the model in diverse scenarios.
